# Large-Scale Integration of Single-Cell RNA-Seq Data Reveals Astrocyte Diversity and Transcriptomic Modules across Six Central Nervous System Disorders

**DOI:** 10.3390/biom13040692

**Published:** 2023-04-19

**Authors:** Zhenwei Qian, Jinglin Qin, Yiwen Lai, Chen Zhang, Xiannian Zhang

**Affiliations:** 1 School of Basic Medical Sciences, Beijing Key Laboratory of Neural Regeneration and Repair, Advanced Innovation Center for Human Brain Protection, Capital Medical University, Beijing 100069, China; 2 Chinese Institute for Brain Research, Beijing 102206, China; 3 State Key Laboratory of Translational Medicine and Innovative Drug Development, Nanjing 210000, China

**Keywords:** astrocytes, CNS disorder, scRNA-seq, spatial transcriptomics, transcriptomic modules, cell–cell communications

## Abstract

The dysfunction of astrocytes in response to environmental factors contributes to many neurological diseases by impacting neuroinflammation responses, glutamate and ion homeostasis, and cholesterol and sphingolipid metabolism, which calls for comprehensive and high-resolution analysis. However, single-cell transcriptome analyses of astrocytes have been hampered by the sparseness of human brain specimens. Here, we demonstrate how large-scale integration of multi-omics data, including single-cell and spatial transcriptomic and proteomic data, overcomes these limitations. We created a single-cell transcriptomic dataset of human brains by integration, consensus annotation, and analyzing 302 publicly available single-cell RNA-sequencing (scRNA-seq) datasets, highlighting the power to resolve previously unidentifiable astrocyte subpopulations. The resulting dataset includes nearly one million cells that span a wide variety of diseases, including Alzheimer’s disease (AD), Parkinson’s disease (PD), Huntington’s disease (HD), multiple sclerosis (MS), epilepsy (Epi), and chronic traumatic encephalopathy (CTE). We profiled the astrocytes at three levels, subtype compositions, regulatory modules, and cell–cell communications, and comprehensively depicted the heterogeneity of pathological astrocytes. We constructed seven transcriptomic modules that are involved in the onset and progress of disease development, such as the M2 ECM and M4 stress modules. We validated that the M2 ECM module could furnish potential markers for AD early diagnosis at both the transcriptome and protein levels. In order to accomplish a high-resolution, local identification of astrocyte subtypes, we also carried out a spatial transcriptome analysis of mouse brains using the integrated dataset as a reference. We found that astrocyte subtypes are regionally heterogeneous. We identified dynamic cell–cell interactions in different disorders and found that astrocytes participate in key signaling pathways, such as NRG3-ERBB4, in epilepsy. Our work supports the utility of large-scale integration of single-cell transcriptomic data, which offers new insights into underlying multiple CNS disease mechanisms where astrocytes are involved.

## 1. Introduction

Astrocytes, one of the most abundant glia cell types in the central nervous system (CNS), have been implicated in the pathogenesis of multiple CNS disorders [1]. Accumulating studies suggest that “reactive” astrocytes have a pathogenic role in both acute and chronic disorders, including Alzheimer’s disease (AD), Parkinson’s disease (PD), Huntington’s disease (HD), multiple sclerosis (MS), and behavioral neuropsychiatric disorders [1,2,3]. Their functions include remodeling of regular physiological processes including metabolism, synapse maintenance, neuroinflammatory regulation, blood–brain barrier (BBB) integrity, and structural supports. Many studies link reactive astrocytes to inflammatory reactions to dying cells, immune cell dysfunction, and pathogenic proteins, including amyloid-β (Aβ), tau aggregation, and alpha-synuclein (αSyn) [3,4,5]. It is worth emphasizing that the tripartite synapse concept proposes that the communication between pre- and post-synaptic neurons is modulated by astrocytes, which constitute the third component of the synapse [6]. Astrocytes are key players in this process, as they modulate synaptic transmission and plasticity through various mechanisms, including the uptake and release of neurotransmitters and the modulation of synaptic signaling pathways [7]. The loss of astrocytic synaptic regulatory functions has been associated with the development of numerous neurological disorders, such as AD, PD, and epilepsy (Epi), underscoring the crucial role of these cells in maintaining proper brain function [7,8]. Therefore, understanding the intricate interactions between neurons and astrocytes in the regulation of synaptic function is crucial for elucidating the underlying mechanisms of brain function and dysfunction.

Among these investigations, high-throughput single-cell RNA sequencing (scRNA-seq) has been the leading method to characterize the cellular heterogeneity in neurological disorders, and its adoption has led to the generation of a plethora of datasets [9,10,11,12]. However, due to the inaccessibility of human brain specimens, most studies focus on a certain CNS disease from a specific brain region [9,13,14]. Moreover, astrocytes were typically underestimated in previous studies, accounting for just 3–18% of nuclei [11]. Therefore, astrocyte heterogeneity and reactivity are not fully demonstrated, leaving many unanswered questions. For example, what is the global landscape of astrocytes, what is the regional specificity of astrocytes, what are their transcriptomic modules, how are these modules regulated, and what intercellular signaling pathways are disturbed among different CNS diseases. We hypothesized that a single study may not be able to provide large enough samples to definitively characterize physiologically relevant yet lowly abundant astrocyte subpopulations.

Large-scale data integration can capture the comprehensive phenotypic features at single-cell resolution to empower the understanding of disease mechanisms [11,15,16]. One of the major issues in integrating a high-quality reference map from scRNA-seq datasets has been the reduction of technical bias and batch effects [17,18]. Recent developments in computational methods have addressed these issues and allowed the generation of large-scale reference atlases [19]. To portray the complete landscape of astrocytes in both physiological and pathological conditions, we gather and integrate large-scale scRNA-seq datasets from extensive human CNS disorders. We harmonize in total 302 human samples of 6 diseases from 15 studies spanning nearly 1 million cells. We identified eight astrocyte subtypes and explored their distribution specificities among brain regions. Meanwhile, we identified seven common expression modules from astrocytes, and module analysis exhibited pronounced heterogeneity across disorders at the transcriptomic level. We also validated these modules at the protein level by using a mass spectrometry dataset [20]. We further explored ligand-receptor signaling pathways and identify dynamic cell–cell interactions in different disorders, thus offering new insights into underlying CNS disease mechanisms.

## 2. Materials and Methods

### 2.1. Data Collection

We collected single-cell transcriptomic, spatial transcriptomic, and proteomic datasets originating from 17 well-generated studies published from June 2019 to March 2022. Some of these samples were donated by healthy individuals, and some were taken from autopsy tissues. In single-cell transcriptome data, except for Wheeler2020, which used scRNA-seq, all others used single-cell nuclear sequencing (snRNA-seq), which ensured the stability of the sequencing method. Similarly, except for Chancellor2021, which used the inDrops sequencing platform, the rest of the data were sequenced using the 10x Genomics platform. The age of the patients we collected ranged from 24 to 102 years old. As our study covers multiple brain regions, even the cortex alone includes more fine-grained subregions such as occipital, occipitotemporal, somatosensory, entorhinal, and dorsolateral cortexes. However, since some of the papers did not specify the exact brain region, we grouped them into four categories, cortex (CTX), hippocampus (HIP), white matter (WM), and basal ganglia (BG), for the analysis. The basal ganglia mainly include the three brain regions of caudate, putamen, and substantia nigra. We named the original brain region sources as ‘source.tissue’, and the new classification is annotated as ‘current.tissue’ in the paper. See Table S1 for detailed information and metadata.

### 2.2. Preprocessing and Quality Control of Single-Cell RNA-Seq Data 

We downloaded all raw scRNA-seq matrix data from different sources (Table S1). We followed Seurat’s guide and processed the unique molecular identifier (UMI) count matrix using the R package Seurat (version 4.1.0) [21]. Several previous scRNA-seq studies have shown that dead cells and cell debris are excluded from datasets containing cells with <300 genes, >50,000 UMIs, or 5% mitochondrial genes [22,23]. We followed these criteria above. The final filtered matrix contained 990,317 cells and 36,590 genes.

### 2.3. Normalization and Initial Dimension Reduction of scRNA-Seq Data 

After preprocessing and quality control, we merged all datasets using the Seurat::merge function. The UMI count matrix was log normalized, and the 3000 most variable features were identified. The linear dimensionality was reduced using the RunPCA function in Seurat. For graph-based clustering, we used the first 30 principal components determined by the ElbowPlot function, which displayed the *p*-value distribution for each principal component. Our initial results from K-nearest neighbor clustering, using FindNeighbors and FindClusters with the resolution = 0.2, and UMAP clustering, using RunUMAP with dim parameter set to 20, were 53 clusters. We found a significant batch effect in the merged datasets, so we performed a subsequent correction for the batch effect.

### 2.4. Batch Correction, Clustering, and Annotation of scRNA-Seq Data

To remove batch effects and non-biological technical biases, the Harmony algorithm in Seurat was used. We have used default values for the other parameters of Harmony [19]. This was followed by dimensional reduction using RunUMAP with dim parameter set to 20. Clustering was identified using the functions FindNeighbors and FindClusters with resolution 0.1. After integration, the batch effects were satisfactorily removed with no notable separation across different platforms or conditions. Raw clustering generated a total of 28 clusters. Major cell types were annotated based on the expression of known markers, i.e., *GFAP* and *AQP4* for astrocyte; *ABCB1* and *FLT1* for endothelial cells; *RBFOX3* and *GRIN1* for neurons; *CAMK2A* and *CBLN2* for excitatory neurons; *GAD1* and *GAD2* for inhibitory neurons; *DOCK8* and *CSF1R* for microglias; *MBP* and *PLP1* for oligodendrocytes; *PDGFRA* and *VCAN* for OPCs; and *PDGFRB* for pericytes.

### 2.5. Subcluster Analysis of Astrocytes

We re-clustered astrocytes (the analysis procedure is the same as above) and identified eight transcriptionally unique subpopulations. To identify the enriched genes in each astrocyte subtype, the FindAllMarkers function (log2FC > 0.25, using a Wilcoxon rank-sum test, adjusted *p*-value < 0.05 using the Bonferroni correction) was used to determine unique and/or highly enriched DEGs in one cluster compared to all other clusters. These cluster-specific features were used as reference genes of GO pathway analysis with the R package clusterProfiler (version 4.2.2, accessed on 11 April 2023) [24]. Data were visualized using Seurat package functions, including DimPlot, FeaturePlot, DotPlot, and DoHeatmap.

### 2.6. The Signature Scores for General Characteristics of Astrocytes

We assessed signature gene scores of astrocytes using the AddModuleScore function in Seurat. These genes originate from previously published studies including PR, pan-reactive astrocytes (*ASPG*, *S1PR3*, *CXCL10*, *VIM*, *GFAP*, *CD44*, *CP*, *OSMR*, *TGM1, FKBP5*, *TIMP1*, *HSBP1*, and *STEAP4*); A1, A1-reactive astrocytes (*C3*, *SRGN*, *GBP2*, *PSMB8*, *AMIGO2*, *FBLN5*, and *SERPING1*); A2, A2-reactive astrocytes (*EMP1*, *CD14*, *CD109*, *PTX3*, *STAT3*, *PTGS2*, *TM4SF1*, *CLCF1*, *SPHK1*, *B3GNT5*, *S100A10*, and *SLC10A6*); HM, homeostasis (*SLC1A2*, *SLC1A3*, *GLUL*, *SLC6A11*, *NRXN1*, *CADM2*, *PTN*, and *GPC5*)*;* and PC, phagocytosis (*MEGF10* and *MERTK*) [25,26,27].

### 2.7. Analysis of Spatial Transcriptomic Data

We downloaded the recently published 10x Visium dataset from whole hemisphere saline or LPS-injected mice (N = 3 per condition) (data available at the Gene Expression Omnibus repository: series GSE165098) [10]. Data were analyzed using Seurat (version 4.1.0) [21]. In short, each section was normalized using SCTransform and merged based on variable features. Data were visualized using the SpatialFeaturePlot function in Seurat.

### 2.8. Regional Annotation of the Mouse Brain

We assessed module scores of classical region-specific genes using the AddModuleScore function in Seurat. These modules include cortex (CTX; *Mef2c*, *Mef2d*), hippocampus (HIP; *Cst3*, *Glul*), white matter (WM; *Plp1*, *Mbp*, and *Mog*), and ventricle modules (*Ccdc153*, *Foxj1*, *Enkur*, and *Ttr*) [28,29,30,31,32]. The midbrain (MB) module gene list was obtained from the MSigDB database (https://www.gsea-msigdb.org/gsea/msigdb/, accessed on 11 April 2023).

### 2.9. Mouse Gene Signatures for Each Astrocyte Cluster

To highlight clusters identified in our single-cell RNA-seq data, we defined gene signatures for each astrocyte cluster using the top 50 marker genes (genes ranked by log2FC) from our earlier enriched genes in each astrocyte subtype (see Subcluster Analysis of Astrocytes and Table S2). To explore the expression of these gene signatures in the mouse dataset as well, we created mouse gene signatures comprising one-to-one orthologs of the human module genes, which were identified using the biomaRt R package (version 2.50.3) [33]. Signature scores were calculated using the AddModuleScore function in Seurat.

### 2.10. Identification of Astrocyte Transcriptomic Modules

The samples with less than 100 astrocytes were excluded. For each of the 294 samples with at least 100 astrocytes, we used non-negative matrix factorization (NMF) to extract 7 co-expression submodules with the NNLM R package (version 0.4.4) (https://github.com/linxihui/NNLM, accessed on 11 April 2023). The specific steps are as follows. We first filtered DEGs of each astrocyte cluster (log2FC > 0.6, adjusted *p*-value < 0.05). We used these genes to construct a normalized expression matrix (genes x cells) for each sample. If the number of astrocytes per sample was greater than 300, we set the number of decomposition rank to 8, and if it was between 100 and 300 then it was set to 6, which resulted in a total of 1556 submodules across the 294 samples. The 1556 submodules were compared by hierarchical clustering using the Pearson correlation coefficient over all gene scores as a distance metric. Based on the clustering dendrogram and cutree function in R, seven clusters of modules were identified manually. We used the top 20 genes of each module to define the module. For a single cell, if it expressed ≥70% of the genes within a given module, it was considered as a cell expressing the activated module, namely, a module cell. 

### 2.11. Functional Analysis of Gene Modules

To functionally describe the astrocyte expression module and non-module cell subtypes, we performed the pathway analysis based on the hallmark and gene ontology gene sets of the MSigDB database and estimated the pathway activity of individual cells using the gene set variation analysis (GSVA) package (version 1.42.0) with standard settings [34]. To assess the differential activities of pathways between module cells and non-module cells, activity scores were contrasted for each cell group using the Limma package (version 3.50.1) [35].

### 2.12. The Interaction Network of the M2 and M4 Modular Genes

We used the top 50 M2 and M4 modular genes to construct the interaction networks by STRING analysis (https://string-db.org, accessed on 11 April 2023) and used Cytoscape (version 3.9.1) to visualize the networks.

### 2.13. Differential Expression Analysis and PCA of Different Disorders

To avoid the spatial bias, the DEG analysis was firstly conducted between disease and controls from the same brain region. The number of unique and overlapping up- and down-regulated DEGs among different disorders are shown using UpsetR (version 1.4.0) [36]. We used genes shared in at least three out of six disorders to construct interaction networks by STRING analysis (https://string-db.org, accessed on 11 April 2023) and used Cytoscape (version 3.9.1) to visualize the networks. PCA was performed based on the top 200 DEGs for each disorder using the scatterplot3d package (version 0.3-41).

### 2.14. Proteomics Dataset Analysis

We downloaded a recently published TMT-MS dataset, which has been normalized [20]. We performed strict quality control and removed proteins and samples containing missing values. After data processing and quality control, a total of 8619 proteins were retained for downstream analysis. We used the top 20 genes of each module to construct protein co-expression modules. At least more than half genes of each module, except M7, consisting of five proteins were paired, but the five proteins were highly correlated with cilium and could greatly represent the M7 module function. We then calculated the corresponding protein abundance scores for each module and assessed correlations within modules. Analysis of modular dependency at the protein level was estimated using Spearman correlations. Correlation coefficients and *p*-values were indicated by a heatmap.

### 2.15. Ligand-Receptor Analysis with CellChat 

Ligand-receptor analysis and visualization were performed using CellChat (version 1.1.3) [37]. CellChat offers reliable quantitative inference, analysis, and visualization of the intercellular communication network based on previously identified ligand/receptor pairings and other signaling cofactors. To dissect this global alteration in different disorders, we first calculated the relative information flows for each signaling pathway using the compareInteractions function, which is defined as the total communication probability among all the pairs of cell groups in the communication network. We also showed key signaling pathways using the netVisual_bubble function. Default values were used for the parameterization of each step.

## 3. Results

### 3.1. Large-Scale Unbiased Integration of Human scRNA-Seq Dataset

**Figure 1:**
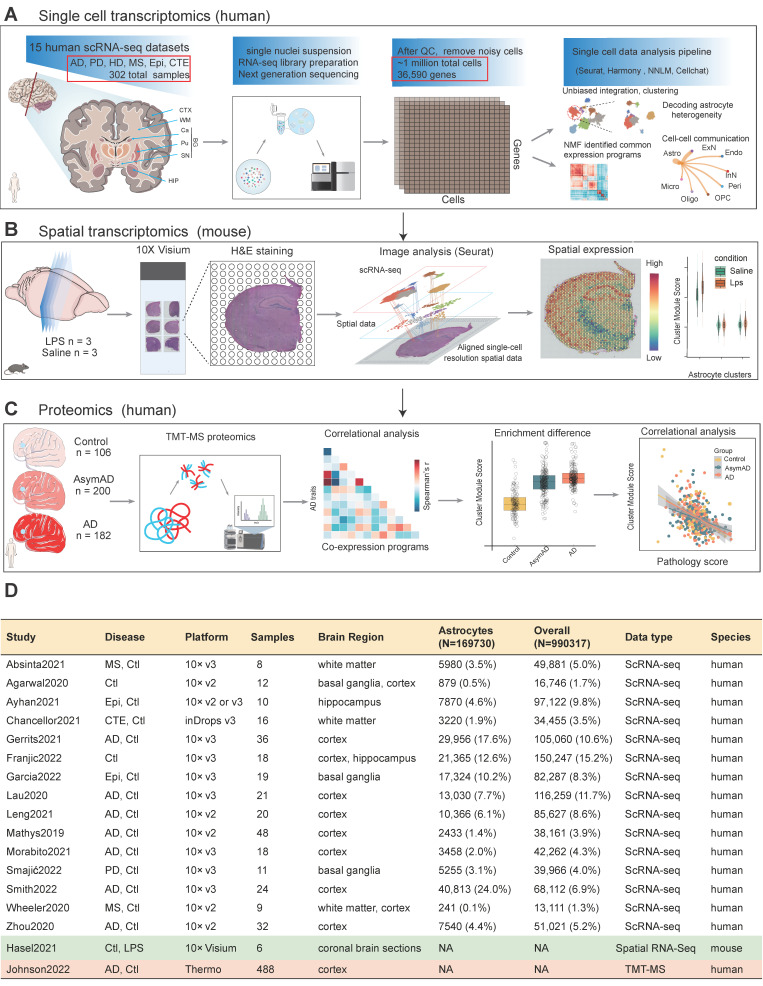
Overview outline of astrocyte dataset categories and analysis pipeline. (**A**) Workflow of data generation, integration, and annotation for the human brains’ single-cell transcriptome datasets. (**B**) Spatial transcriptomics profiling workflow of mouse brains. (**C**) Processing workflow of the TMT-MS proteomics dataset on control, asymptomatic AD (AsymAD), and AD brain tissues. (**D**) Metadata table for all datasets included in this study. Abbreviations: AD, Alzheimer’s disease; PD, Parkinson’s disease; HD, Huntington’s disease; MS, multiple sclerosis; Epi, epilepsy; CTE, chronic traumatic encephalopathy; AsymAD, asymptomatic AD; scRNA-seq, single-cell RNA-sequencing; spatial RNA-seq, spatial RNA-sequencing; TMT-MS, tag mass tag spectrometry.

**Figure 2:**
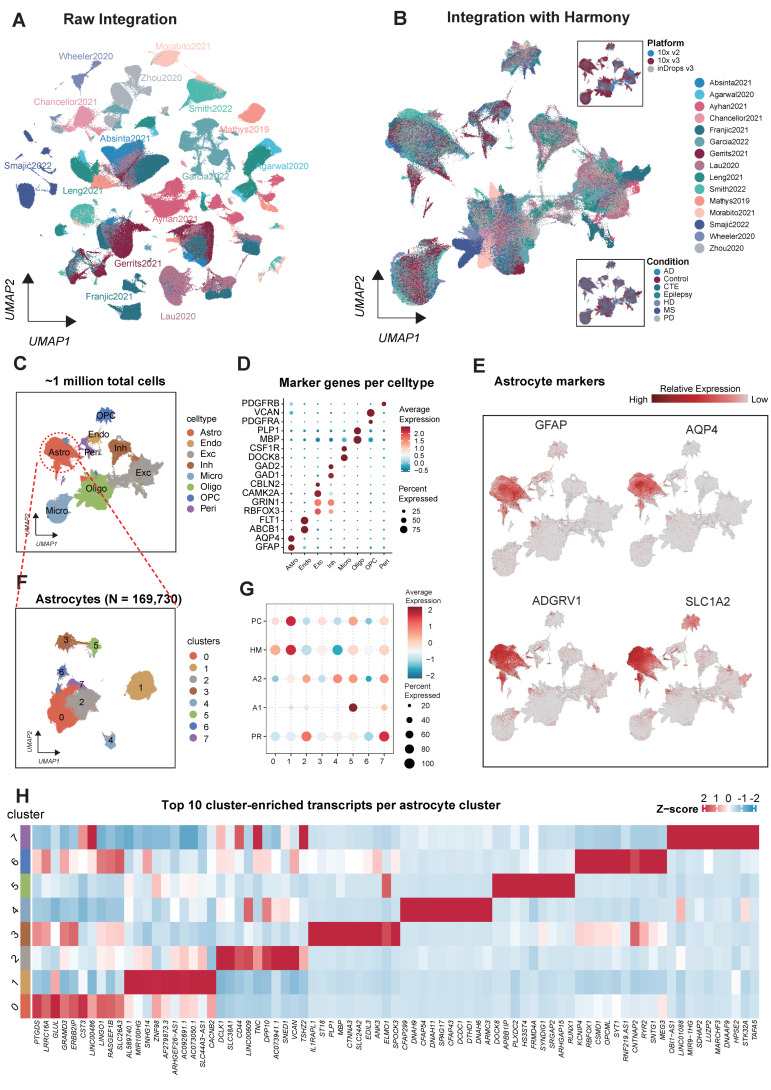
Large-scale integration of 302 single-cell RNA-seq samples reveals astrocyte subtypes in the human brain. (**A**) UMAP plot of the combined datasets before batch correction, colored by dataset source. (**B**) UMAP plot of integrated dataset after batch correction with Harmony, colored by dataset source. The corner insets are colored by scRNA-seq platforms (up) and disease types (down). (**C**) UMAP plot colored by major cell types in the human brain. (**D**) Expression of canonical marker genes of major cell types. Dot plot showing the expression level (color scale) and the percent of cells expressing (dot size) marker genes across cell types. (**E**) UMAP plots of all cells colored by expression levels of four classical astrocyte markers. (**F**) A total of 169,730 astrocytes were re-clustered into 8 astrocyte subtypes (cluster 0−7). (**G**) General characteristics of 8 subtypes using relative expression strength of 5 known astrocyte programs: phagocytosis (PC); homeostasis (HM); A1− and A2−reactive astrocytes (A1, A2); and pan-reactive astrocytes (PR). (**H**) Heatmap showing the normalized average expression of the top 10 enriched genes for each cluster.

The overall strategy includes collecting human scRNA-seq, spatial transcriptomic, and proteomic datasets to avoid the limitations by single methodology (Figure 1A–C). Briefly, we performed quality control and integration for scRNA-seq datasets from human CNS disease tissues (Figure 1A). To provide a high-resolution reference map for their regional preferences, a mouse spatial-transcriptomes dataset was included (Figure 1B). To validate these astrocyte subtypes and modules at the protein level, we used a published tandem mass tag mass spectrometry (TMT-MS) dataset in AD patient brain tissues (Figure 1C). In total, we curated 302 publicly available human brain scRNA-seq datasets that were generated by different platforms (10x v2/3 and inDrops v3) from June 2019 to March 2022 (Figure 1D). They originated from six CNS diseases, known as AD, PD, HD, MS, epilepsy (Epi), and chronic traumatic encephalopathy (CTE). After removing ambient RNA signatures and removing cells of low quality (Supplementary Figure S1A), we merged the datasets to perform preliminary single-cell transcriptome analysis. The resulting dataset consisted of 990,317 cells and nuclei (Figure 2A and Table S1). Due to differences in the specimen sources, experimental conditions, and library preparation methods, substantial batch effects were evident in dimensionally reduced visualizations of these data (Figure 2A). After Harmony integration, the batch effects were satisfactorily removed with no notable separation across different platform or conditions (Figure 2B). For integrated datasets, network-based clustering analysis revealed 28 cell clusters, which were further manually categorized into 8 major cell types using canonical marker genes including astrocytes (Astro), endothelial cells (Endo), excitatory (ExN) and inhibitory (InN) neurons, microglia (Micro), oligodendrocytes (Oligo), oligodendrocyte progenitor cells (OPCs), and pericytes (Peri) (Figure 2C,D). Each dataset contained all the cell types (Supplementary Figure S1B,C). Thus, we have completed the integration of the datasets without bias and identified the main cell types for downstream analysis.

### 3.2. Molecularly and Functionally Distinct Astrocyte Subtypes Are Identified

In total, 169,730 astrocytes were identified by showing high expression of markers such as *ADGRV1*, *AQP4*, *SLC1A2*, and *GFAP* (Figure 2E). We sought to determine whether the increased cell numbers enabled us to detect novel subpopulations that had previously been missed. Subcluster analysis of astrocytes generated eight transcriptionally unique subpopulations (Figure 2F–H). The biological relevance of each subtype was checked using several published functional gene signatures including phagocytosis (PC), homeostasis (HM), and A1-, A2-, and pan-reactive astrocytes (PR) [25,26,27] (Figure 2G). Their marker gene sets and functional annotations were listed (Supplementary Figure S3 and Table S2). We found that clusters 0 and 1 both highly expressed homeostasis signatures involved in amino acid transport, transportation across the blood–brain barrier, and positive regulation of synaptic transmission (e.g., *SLC1A2*, *SLC1A3*, and *GLUL*), suggesting their important putative protective roles in maintaining CNS homeostasis. In addition, cluster 0 was also involved in transition metal ion homeostasis (e.g., *MT3*, *FTL*/*FTH1*), and expressed *APOE* and *CLU* involved in amyloid metabolism and clearance [38,39,40]. Interestingly, some lncRNAs, *SNHG14* and *MIR100HG*, were enriched in cluster 1. *SNHG14* has previously been reported to provide neuroprotection by inhibiting inflammatory responses during AD progress [41]. In addition, phagocytosis-associated signatures (*MEGF10*, *MERTK*) were significantly enriched in cluster 1, which has previously been reported as a novel role for astrocytes in mediating synapse elimination to achieve precise neural connectivity [42,43]. Therefore, we hypothesize that there are subtype-specific astrocytes and not all cells perform this phagocytic function in the adult brain.

Pan-reactive astrocyte signatures (e.g., *GFAP* and *CD44*) were enriched in cluster 2 and 7, representing astrocyte activation [44,45]. Cluster 2 highly expressed genes involved in oxidative stress (*FOS*, *JUN*/*JUNB*, *UBC*, and *ID3*) and the extracellular matrix (*TNC*, *TNR*, and *VCAN*) and associated with proteostasis (e.g., *HSP90AA1*, *HSPA1A*, and *HSPB1*, known as chaperones). These reactive genes presenting in the same astrocyte cluster suggest their putative close interaction. Cluster 5 was enriched for inflammation/A1 astrocyte signatures and highly expressed immune response genes including *DOCK8*, *APBB1IP*, *C3*, *SPP1*, *RUNX1*, and *PLXDC2*. Previously published studies have reported complement component 3 (C3) in a specific subtype of reactive astrocytes that respond to inflammation and mediate microglia–astrocyte crosstalk in a range of neurodegenerative diseases such as AD, HD, MS, and amyotrophic lateral sclerosis (ALS) [4,26,46]. Cluster 4 expressed many microtubule-related genes involved in the formation of the cytoskeleton. Interestingly, we found that cluster 3 expressed oligodendrocyte marker genes (*MBP* and *PLP1*) and higher *OLIG2* (oligodendrocyte transcription factor 2) compared with other clusters (*p* = 0.022, Table S2). We adopted the expression of *MBP* in microglia cells as a measurement of background *MBP* levels. We compared the total *MBP* expression level of all astrocyte cells or astrocyte cluster 3 to that of microglia. We found that *MBP* was expressed at much higher levels in astrocytes than in microglia, which indicates that we have ruled out the possibility of acellular free-RNA cross-contamination affecting the properties of astrocytes (Supplementary Figure S2C). Therefore, cluster 3 likely represented OLIG2^+^ astrocyte subtypes involved in ensheathment of neurons [47,48]. Lastly, cluster 6 especially expressed synapse-associated genes (e.g., *SYT1*, *NLGN1*, and *GRIN2B*) and was involved in synaptic maintenance. Together, we identified eight transcriptionally distinct astrocyte subtypes, suggesting that integrated data enabled us to detect novel subpopulations that had previously been missed.

### 3.3. Astrocyte Subtypes Are Ogeneous in Human and Mouse Brains

**Figure 3:**
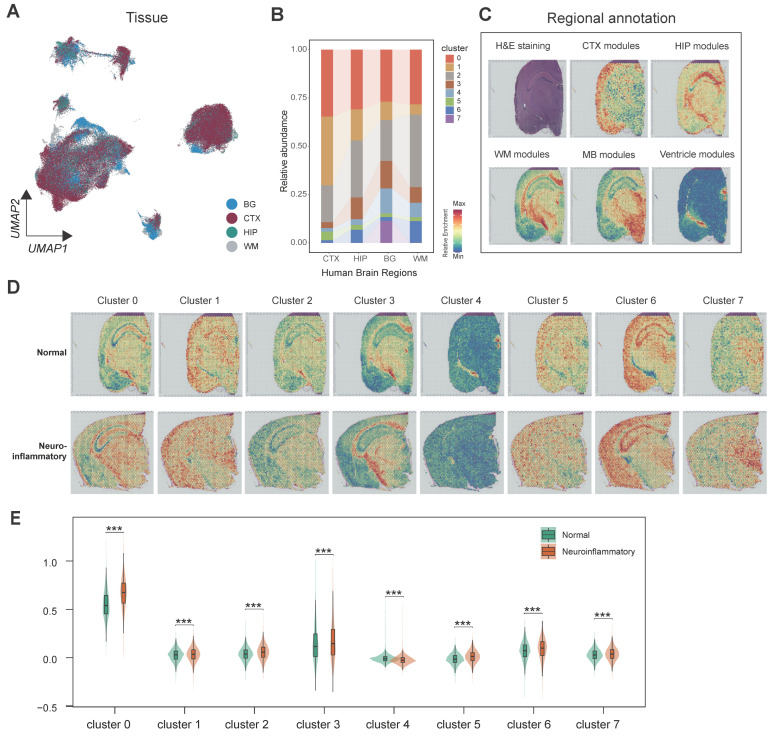
Astrocyte subtypes are regionally heterogeneous. (**A**) UMAP plot of astrocytes, colored by the human brain regions including basal ganglia (BG), cortex (CTX), hippocampus (HIP), and white matter (WM). (**B**) Relative proportions of astrocyte subtypes for samples from different brain regions. BG, N = 40; CTX, N = 209; HIP, N =16; WM, N = 29. (**C**) Identification of brain region locations using module scores of classical region-specific marker genes: CTX, HIP, WM, midbrain (MB), and ventricle modules. (**D**) The highlighted spatial distribution of 8 astrocyte subtype signatures in sections from normal (N = 3) and neuroinflammatory (N = 3) brains using Visium spatial transcriptomics. (**E**) Quantification of signature scores of the top 50 enriched genes for each cluster, across all spots from normal and neuroinflammatory samples. (*** *p* < 0.001, Kruskal–Wallis test).

Recent findings showed astrocytes varied across the CNS regions [10,11,49]. We next aimed to determine whether our diverse astrocyte subgroups are located in various parts of the brain. We found that some astrocyte cluster modules exhibited significantly region-specific expression in the mouse brain, in line with the origin of astrocytes in human brain tissue (Figure 3A). For example, cluster 1 was significantly enriched in the cerebral cortex of humans where *RORB* and *TSHD7A* were enriched, which have previously been reported to show cortex-specific expression in astrocytes and neurons [49,50] (Figure 3B).

In order to examine regional variations in our astrocyte subtypes in the human and mouse brain, we used available spatial transcriptomics datasets [10] (Figure 3C,D). To precisely annotate the spatial transcriptomics datasets, we labeled the brain slices with the expression level of known regional specific markers (Figure 3C) including cortex (CTX; *Mef2c*, *Mef2d*), hippocampus (HIP; *Cst3*, *Glul*), white matter (WM; *Plp1*, *Mbp*, and *Mog*), midbrain (MB, see methods), and ventricle modules (*Ccdc153*, *Foxj1*, *Enkur*, and *Ttr*) [28,29,30,30,31,32] (Figure 3C). We then generated the spatial expression of assembled marker genes for each cluster to estimate the likely location of each astrocyte population (Figure 3D). These cluster signatures have diversified brain region distributions. Cluster 1 signatures are enriched in the cortex (Figure 3D). Notably, we discovered that cluster 6 was considerably enriched in the mouse cortex and hippocampal regions, which is in line with the involved synapse formation function (Figure 3D). The cluster 4 signature was exclusively observed in regions surrounding the 3rd and lateral ventricles of the mice, suggesting that cluster 4 might interact with some factors carried by the cerebrospinal fluid [10,51]. Meanwhile, we also validated the expression of tanycyte markers such as COL23A1 and SLC16A2 in cluster 4, and we did not detect the expression of these two genes in cluster 4 (Supplementary Figure S2B) [52]. Therefore, we excluded the possibility that cluster 4 represents tanycytes. Interestingly, we found that cluster 7 from human basal ganglion (BG) tissues was significantly enriched in MB of the mouse brain. In addition to expressing genes involved in cell–cell and cell–ECM (extracellular matrix) interaction (*TNC*, *CD44*, and *APOE*), cluster 7 also highly expressed *LUZP2* (log2FC = 2.48) compared to other clusters (Table S2). This is consistent with the report that an LUZP2^+^ astrocyte subset exists in the mouse medial amygdala, part of the BG [53]. These results suggested that cluster 7 may represent a novel astrocyte subtype unique to the basal ganglion in human and mouse brains. In addition to the conserved subtypes shared between mouse and human brains, we also found species-specific differences, such as the large proportion of cluster 0 distributed in the cortex of the human brain, which we found difficult to detect in the mouse cortex. This may be due to the lack of interlaminar and varicose projection astrocytes in rodent animals, which has been reported by many studies [54,55].

In addition, we also explored whether neuroinflammation induced by the endotoxin lipopolysaccharide (LPS) affects the expression of module signatures. We found that all the module signatures, except for cluster 4, were significantly upregulated in neuroinflammatory mice compared to normal controls (Figure 3E). Together, we discovered notable and robust brain area preferences for astrocyte clusters that are shared between the human and mouse datasets, such as clusters 1 and 7, indicating that the astrocyte subtypes we found may be preserved between species at the transcriptome level. Meanwhile, we also found differences in the transcriptional profiles of some astrocyte subtypes between human and mouse brains, such as cluster 0, which indicates species-specific variation.

### 3.4. Astrocyte Subtypes Are Partially Associated with CNS Disease

To investigate how astrocytes contribute to disease onset, we preliminarily analyzed the relationship between astrocyte subtypes and disease (Supplementary Figure S4). We found clusters 2 and 7 showed an increase in their relative abundance in PD compared with control groups in the same regional brain. In contrast, clusters 3 and 5 showed a sharp decrease in PD. Furthermore, we found that multiple astrocyte subtypes were involved in the development of epilepsy and multiple sclerosis, such as clusters 1, 2, 3, and 5 in epilepsy and clusters 3 and 4 in MS. Interestingly, we also identified two astrocyte subtypes, clusters 0 and 6, associated only with HD and CTE, respectively, likely indicating that only the sole astrocyte subtype, but not all, is involved in disease progression.

Unfortunately, we could not find statistical differences in AD compared with control groups, which is contrary to previous studies [11,56]. We hypothesized that pathological stimulus does not always lead to quantitative changes in astrocyte subtype compositions but could induce functional changes in the form of interference of gene modules. In addition, we have found that some diseases are associated with multiple astrocyte subtypes, and it remains unclear whether the common or specific functions of these astrocytes are responsible for the development of the disease. Therefore, to overcome these problems, we set out to uncover the prevalent regulatory modules across all subsets of astrocytes. 

### 3.5. Identification of Seven Common Expression Modules of Astrocytes across Control and Disease Tissues

**Figure 4:**
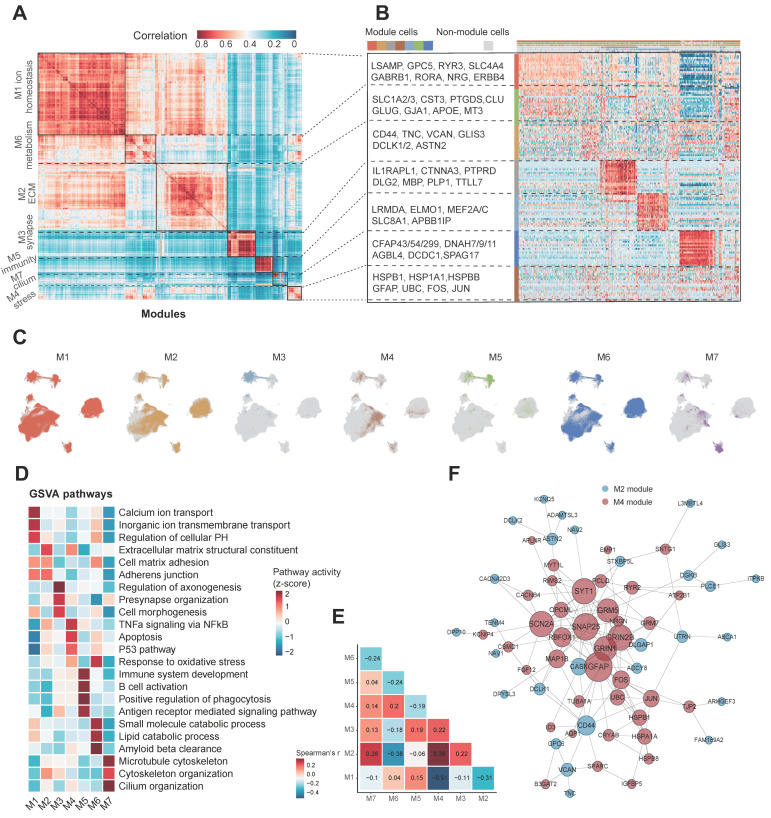
Identification of astrocyte transcriptomic gene modules. (**A**) Heatmap showing pairwise correlations of non-negative matrix factorization (NMF) submodules derived from 294 samples. The resembled submodules across samples are aggregated into 7 modules (M1−M7). (**B**) Heatmap showing expression of marker genes within each module across single cells. Colors above columns correspond to cell state. (**C**) The distributions of each expression module. Cells expressing ≥70% of genes in each module are defined as module cells. (**D**) Heatmap showing pathway activity differences between module cells and non-module cells for each module scored by GSVA. Each column is normalized by z−score to indicate the relative pathway activities. (**E**) Heatmap showing Spearman correlation coefficients assessing for each pair of modules (rows, columns) if they are co-occurrent (≥0.3, red, positive correlation) or exclusive (≤−0.3, blue, negative correlation). (**F**) The interaction networks for the M2 ECM and M4 stress modular genes (blue, M2 modular genes; red, M4 modular genes). Node size is the number of links; edges represent interactions between nodes.

We used non-negative matrix factorization (NMF) for 294 human brain scRNA-seq datasets to uncover coherent sets of genes that were preferentially co-expressed by subsets of astrocytes. We identified seven expression modules (M1–M7) with different functions and cell statuses (Figure 4A,B; Table S3) and defined cells expressing ≥70% of genes in each module as module cells (Figure 4C). We selected the most activated pathways in the module cells by comparison with non-module cells to analyze their functions using GSVA analysis (Figure 4D). Despite the fact that the number of modules and previously identified clusters are similar, they are not directly corresponded, as many modules tend to span across multiple clusters. Specifically, M1, M2, M6, and M4 modules are widely expressed with different strengths, suggesting that they are necessary for most astrocyte subtypes to perform basic functions (Figure 4C). The M1 ion homeostasis module was characterized by the expression of genes associated with ion transport (e.g., *GPC5*, *RYR3*, *SLC4A4*, and *GABRB1*). M1 module cells were involved in calcium ion transport, inorganic ion transmembrane transport, and regulation of cellular PH pathways (Figure 4D). The M6 metabolism module consisted of metabolism-associated genes (e.g., *CLU*, *GLUG*, *GJA1*, *APOE*, and *MT3*), which comprise two AD-risk genes (*APOE*, and *CLU*). M6 module cells also displayed small molecule catabolic process, lipid catabolic process, and amyloid beta clearance (Figure 4D). These results suggest M6 plays a critical role in AD pathogenesis by metabolically interacting with neurons [57]. The M2 ECM module had increased expression of ECM-associated genes (e.g., *CD44*, *TNC*, and *VCAN*) and protein kinases (*DCLK1/2*) that were involved in radial migration and axon growth of cortical neurons and associated with neurodevelopmental and neuropsychiatric disorders [58,59]. Additionally, *GLIS3* in the M2 module has been studied to influence the glioma cells’ invasion, migration, and proliferation and upregulate the NF-κB signaling pathway [60]. The M4 stress module is a known signature of reactive astrocytes highly expressed in stress/inflammatory genes such as heat shock proteins (*HSPB1*, *HSP1A1*, and *HSPBB*) and cellular stress (e.g., *GFAP*, *UBC*, *FOS*, and *JUN*). M4 stress cells showed activation of the response to oxidative stress, P53 pathway, apoptosis, and TNFα signaling (Figure 4D). 

In contrast, other modules were expressed in different astrocyte clusters in a special-cluster manner, such as M3 in cluster 3, M5 in cluster 5, and M7 in cluster 4 (Figure 4C). The M3 synapse module was characterized by high expression of genes involved in synapse organization (e.g., *IL1RAPL1*, *CTNNA3*, *PTPRD*, and *DLG2*) and ensheathment of neurons (e.g., *MBP*, *PLP1*, and *ST18*), in line with displaying regulation of axonogenesis and presynaptic organization pathways (Figure 4B,D). The M5 immunity module consisted of immune response genes (*LRMDA*, *ELMO1*, *MEF2A/C*, *SLC8A1*, and *APBB1IP*) and performed immune activation functions, including positive regulation of phagocytosis and the antigen receptor-mediated signaling pathway [61,62]. The M7 cilium module had overexpressed genes related to the cilium and microtubule, such as cilium proteins (e.g., *CFAP43/54*, *DNAH7/9*, *HYDIN*, and *RFX3*) and microtubule proteins (e.g., *AGBL4*, *DCDC1*, and *SPAG17*). Together, we identified seven co-expression modules of astrocytes from multiple samples, which perform specific functions.

### 3.6. Identification of Dependency among the Expression Modules

We next explored how these modules are co-regulated in pairwise using Spearman correlation analysis (Figure 4E). We identified one significant co-occurring module pair as well as three mutually exclusive module pairs (r ≥ 0.3 or ≤ −0.3, *p* < 0.05, Figure 4E). The M2 ECM showed the strongest positive correlation with the M4 stress/inflammation (r = 0.36). We found an intense interaction network among these two modular genes, probably reflecting their cooperation and coregulation in multiple processes (Figure 4F). Astrocytes as a source for ECM and cytokines are well known, and there is growing evidence that astrocytes communicate through direct cell–cell contacts or via the extracellular matrix to transmit the activated state to individual ECM-connected cells, which is associated with various neurological diseases [63,64,65,66]. Another study finds that matrix stiffness affects astrocytic activation and phenotypes in an in vitro injury model [67]. Notably, M1 ion homeostasis showed the strongest negative correlation with the M4 stress/inflammation (r = −0.51), which likely reflects that astrocyte reactivity could disrupt ion homeostasis of the brain microenvironment. Together, these results indicate that astrocytes have function-specific co-expression modules, and these modules are highly heterogeneous among astrocyte subtypes.

### 3.7. Brain Region Is a Key Factor for Transcriptome Differences in Astrocytes

**Figure 5:**
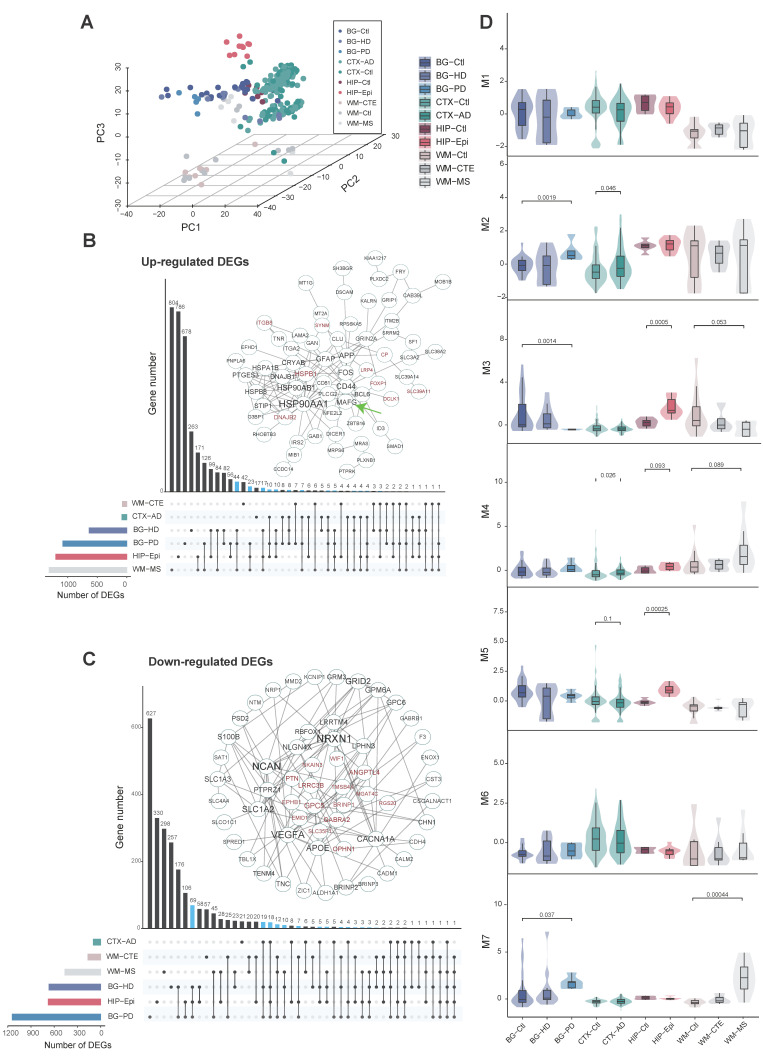
Gene and modular transcriptomic changes across multiple CNS disorders and brain regions. (**A**) Three-dimensional PCA plot for different CNS disorder samples based on disease-associated genes. Samples are grouped by both brain region and disease condition: basal ganglion control (BG-Ctl, N = 24), basal ganglion Huntington’s disease (BG-HD, N = 11), basal ganglion Parkinson’s disease (BG-PD, N = 5), cortex Alzheimer’s disease (CTX-AD, N = 120), cortex control (CTX-Ctl, N = 89), hippocampus control (HIP-Ctl, N = 6), hippocampus epilepsy (HIP-Epi, N = 10), white matter control (WM-Ctl, N = 13), white matter chronic traumatic encephalopathy (WM-CTE, N = 8); white matter multiple sclerosis (WM-MS, N = 8). (**B**) UpSet plot showing unique and overlapping up-regulated DEGs for each CNS disorder. The inset panel demonstrates interaction networks among collectively up-regulated genes in at least three out of six disorders. DEGs in at least four out of six disorders are colored in red. (**C**) A similar analysis for down-regulated DEGs across CNS disorders. (**D**) Boxplots showing the distribution of module scores in all control and disorder groups as described in (**A**). *p*-values are derived from a two-sided Wilcoxon test.

To study whether astrocytes have contextual changes across different CNS disorders, we next analyzed differentially expressed genes (DEGs) and identified both common and disorder-specific transcriptomic changes across different CNS disorders including HD, PD, AD, Epi, CTE, and MS. To avoid spatial bias, the DEG analysis was conducted between disease and controls from the same brain region. In total, we identified 2294 upregulated DEGs and 3407 down-regulated DEGs across all disorders that were presumably driven by disorders (Table S4). Principal component analysis (PCA) of reactivity DEGs was performed to infer the correlation among different diseases (Figure 5A). It was expected that these samples were separated according to disease types. However, the PCA showed near-orthogonal differences, and samples from the same brain region had the closest proximity, indicating that the astrocyte heterogeneity among different brain regions could be stronger than that of disease states. Several recent studies have demonstrated the existence of astrocyte spatial heterogeneity, and astrocytes could produce spatial-specific and subtype-specific responses to inflammatory or pathological stimuli by in situ hybridization and spatial transcriptomics [10,32,68]. Meanwhile, we also found that samples of hippocampus and cortex tissues are closely grouped, suggesting that hippocampus and cortex astrocyte transcriptional levels are similar. However, epilepsy samples of the hippocampus broke away from clusters of the same tissue (Figure 5A), probably reflecting that epilepsy pathology causes greater transcriptional alterations in astrocytes compared to regional factors.

### 3.8. Collectively Up- and Down-Regulated Transcriptomic Changes across Multiple CNS Disorders

We further explored transcriptomic differences across different CNS disorders and found that transcriptional changes associated with astrocyte reactivity are highly heterogeneous in a disorder-specific manner. The number of up-regulated DEGs varies greatly across disorders: MS (1315), epilepsy (1203), PD (1082), HD (639), AD (84), and CTE (76) (Figure 5B). Their functions are annotated using GO analysis (Supplementary Figure S5A and Table S4). Among them, 157 genes were up-regulated in at least 3 out of 6 disorders (Figure 5B and Table S4). They formed an interaction network, indicating their highly connected regulations, which implied strong codependency of pathogenic processes in multiple CNS disorders through intercellular communications [69,70]. Reactive and stress signature genes formed the core of this network, including *GFAP*, *FOS,* and heat-shock genes. This network also suggested that many astrocytic genes are associated with multiple pathological processes including *MAFG*, *CLU*, *APP*, and *ITM2B/C*. Specifically, a recent study identified that *MAFG*-driven astrocytes promote CNS inflammation and repress antioxidant and anti-inflammatory transcriptional modules in MS and its preclinical model, experimental autoimmune encephalomyelitis (EAE) (Wheeler et al., 2020). Here, we further revealed that in addition to MS, HD, and PD, astrocytes also highly express *MAFG*. Given that comparatively less is known about the role of *MAFG* in other CNS diseases, we hypothesize that the same pathological mechanisms may exist in the other two diseases. 

For the down-regulated genes, PD, Epi, HD, and MS had more DEGs compared to AD and CTE disorders (Figure 5C). A total of 178 genes were down-regulated in at least 3 out of 6 disorders. Some of these shared genes include *EPHB1*, *PTN*, *NRP1*, *NRXN1*, and *GABRA2,* which are involved in synapse organization, axonogenesis, and positive regulation of glial cell differentiation (Supplementary Figure S5C and Table S4). The lowered expression levels of some of these transcripts, which have significant potential protective functions in neurodegenerative diseases, might be worrisome. For example, ephrin type-B receptor 1 (*EphB1*) in a mouse ALS model and pleiotrophin (*PTN*) in MS could reduce pro-inflammatory signaling in astrocytes and promote neuronal survival following inflammatory challenge [71,72]. We found that astrocytes in multiple disorders also downregulated pathways associated with calcium channel regulator activity and blood vessel endothelial cell migration, suggesting that astrocytes may be losing important homeostatic and protective functions in disease conditions. Together, we identified both disorder-specific and collective transcriptomic changes across different CNS disorders in astrocytes and further revealed the similarities and differences among CNS disorders. These findings provide a meaningful approach and results for studying associations among diseases.

### 3.9. Gene Module Alterations across Different CNS Disorders

To explore whether there are some associations between the previously constructed modules and the diseases, we firstly calculated co-expressed module scores for the top 20 genes of each module by case status. Then, we compared the module scores between disease and control groups in the same brain region (Figure 5D). Notably, we identified both shared and disorder-specific module changes in different CNS disorders. For instance, the M2 ECM module was significantly increased in PD and AD, suggesting that the ECM performs a general function in both disease processes. Similarly, we found that the M3 synapse module of PD and Epi and the M7 cilium module of PD and MS were also elevated (Figure 5D). In addition to shared modules in multiple disorders, we identified disease-specific modules as well, such as the M4 stress module up-regulated in AD and the M5 immunity module up-regulated in epilepsy (Figure 5D). These results agree with and promote the awareness that glial cells play an important role in epilepsy, which involves inflammation, oxidative stress, and maladaptive myelination [73,74]. Compared to other diseases, we found that AD is most susceptible to the M4 stress/inflammation module. PD in the basal ganglion and AD in the cortex are both more highly expressed in the M2 ECM module, indicating that ECM is significantly associated with pathological processes of them. In summary, we further assessed the association between diseases and co-expressed modules in astrocytes and found some functional modules played an important role in the development of the disease.

### 3.10. Validation of Astrocyte Modules in AD Proteomic Dataset

**Figure 6:**
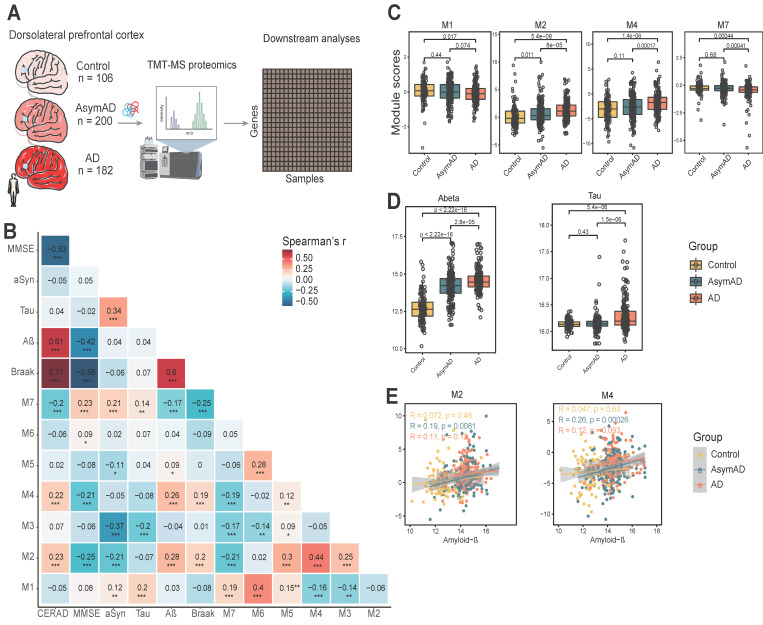
Astrocyte transcriptomic modules were validated at the protein level. (**A**) Workflow of TMT-MS-based proteomics on dorsolateral prefrontal cortex (DLPFC) tissues from control (N = 106), asymptomatic AD (AsymAD, N = 200), and AD human brains (N = 182). (**B**) Heatmap shows module relatedness, and modules were correlated with neuropathological and cognitive traits. Correlation coefficients were asserted based on Spearman correlation (red, positive correlation; blue, negative correlation). * *p* < 0.05, ** *p* < 0.01, and *** *p* < 0.001. Amyloid plaque load (CERAD score), tau neurofibrillary tangle burden (Braak stage), cognitive function (Mini-Mental Status Examination, MMSE), and mass spectrometry molecular measurements (Aβ; tau, the tau microtubule-binding region; αSyn). (**C**) Boxplots showing module score levels by case status. *p*-values are derived from a two-sided Wilcoxon test. (**D**) Boxplots showing Aβ and tau levels by case status. *p*-values are derived from a two-sided Wilcoxon test. (**E**) Aβ levels were correlated with M2 and M4 module scores by case status. Correlation coefficients were asserted based on Spearman correlation, colored by case status.

To confirm our transcriptome-based results at the protein level, we re-analyzed the tandem mass tag mass spectrometry (TMT-MS) dataset in AD [20]. We included a total of 488 dorsolateral prefrontal cortex tissues including control (N = 106), asymptomatic AD (AsymAD, N = 220), and AD (N = 182) groups (Figure 6A). We then calculated the corresponding protein abundance scores for each module and assessed correlations within modules (Figure 6B). Surprisingly, there was a stronger correlation between some modules at the protein level compared to the RNA level, such as M2/M4 (r = 0.44) and M1/M6 modules pairs (r = 0.4). These results again demonstrated that ECM and inflammation indeed are functionally linked. Interestingly, we found some modules showed an opposite relationship, such as M2/M5, M2/M7, and M5/M6, suggesting that significant differences between protein and RNA co-expression exist [20,75].

The module scores were correlated with amyloid plaque load (CERAD score), tau neurofibrillary tangle burden (Braak stage), cognitive function (Mini-Mental Status Examination, MMSE), and mass spectrometry molecular measurements (Aβ; tau, the tau microtubule-binding region; αSyn) (Figure 6B). We observed that the M2 and M4 modules showed the strongest AD trait correlations (CERAD score and Aβ r > 0.2, MMSE r < −0.2). We also found some pathology-specific modules. For example, the M1 ion homeostasis and M7 cilium modules showed a passive correlation with tau and αSyn proteins. However, the correlation between M7 and Aβ was opposed.

We associated our astrocyte modules with diagnostic classifications of AD (Figure 6C). In general, most modules were increased or decreased consistently with AD progressions in the AsymAD group, indicating that these modules could reflect pathophysiologic processes that begin early in the preclinical phase of AD. Notably, the M2 ECM module was more significantly increased in AsymAD compared to control. The accumulation of β-amyloid occurred in early stages of AD (Figure 6D), which was consistent with previous reports [76]. To further investigate susceptibility to the early stages of AD, we correlated each module score to neuropathological traits by case status (Figure 6E). We found that M2 and M4 showed the positive correlation with Aβ only in AsymAD, which suggested that the two network modules could sensitively perceive the pathological changes of the disease in the early stages of AD (Figure 6E). Overall, we again validated these co-expression modules here at the protein level and screened for key modules, such as the astrocytic M2 ECM module, that may have early diagnostic value for AD.

### 3.11. Integrated scRNA-Seq Data Show Alterations in Cell–Cell Communications in Different Disorders

**Figure 7:**
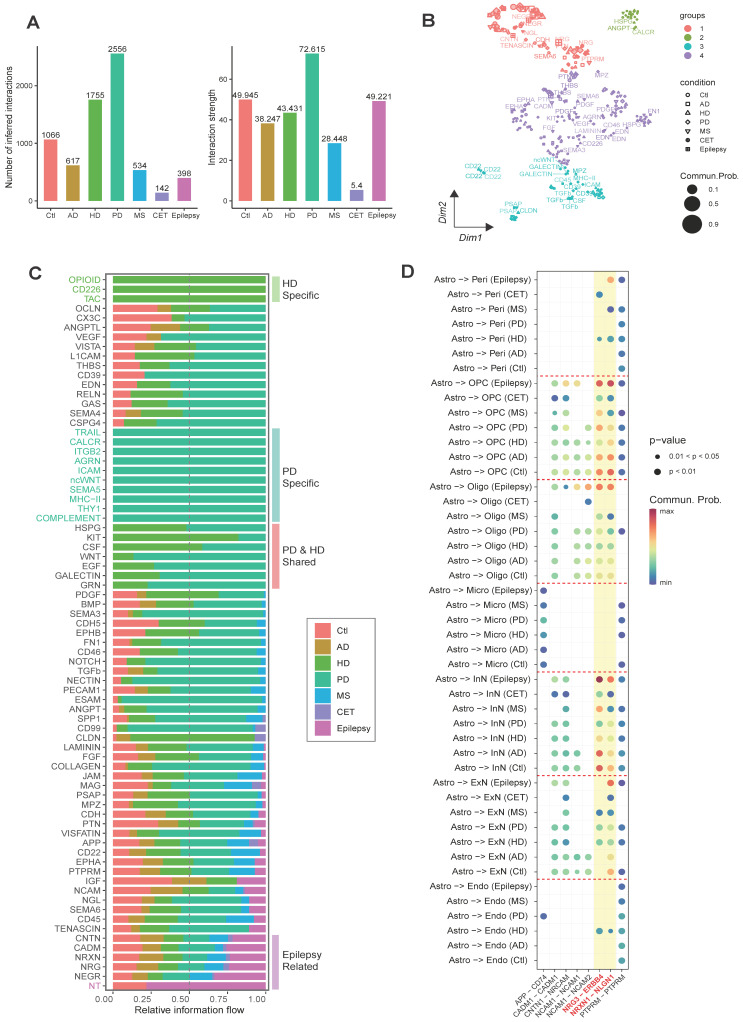
Identification of CNS disorder-specific cellular signaling changes. (**A**) Barplot of the number of inferred interactions and interaction strength among all cell subpopulations in different disorders. (**B**) Jointly projecting and clustering signaling pathways from different disorders into a shared two-dimensional manifold according to their functional similarity. Different symbols represent the signaling networks from different disorders. Each symbol represents the communication network of one signaling pathway. The symbol size is proportional to the total communication probability. Different colors represent different groups of signaling pathways. (**C**) Barplot of all significant signaling pathways showing their relative information flow across disorders. (**D**) Dotplot comparing the significant ligand-receptor pairs among different CNS disorders, which contribute to the signaling from astrocytes to other cell types. Dot color reflects communication probabilities and dot size represents computed *p*-values. Empty space means the communication probability is zero. *p*-values are computed from a one-sided permutation test.

Astrocytes intensively interact with surrounding cell types through signaling crosstalks, which are significantly perturbed in diseases. We explored the changes in intercellular signaling in different diseases with CellChat [37]. We first calculated the number of inferred interactions and interaction strength among all cell subpopulations and found significant differences in cellular communication among different disorders (Figure 7A). We used eight common cell populations among different disorders to perform functional similarity analysis. As the result, the signaling pathways associated with inferred networks from different disorders were mapped onto a shared two-dimensional manifold and clustered into groups. We identified four pathway groups (Figure 7B). Groups 1, 2, and 4 were dominated by growth factor pathways such as PDGF, NEGT, NRG, VEGF, and ANGPTL, while group 3 dominantly contained immune-related pathways such as ncWNT, CD45, ICAM, and MHC-II. We then compared relative information flows for each signaling pathway among different disorders and found significant disease-specific cellular communications (Figure 7C). For example, three signaling pathways were highly active in HD, which were involved in inflammatory and immune responses, such as OPIOID, CD226, and TAC. Ten pathways were specifically active in PD, including known immune signals such as TRAIL, ITGB2, ncWNT, MHC-II, ICAM, and COMPLEMENT. Interestingly, the neurotrophin (NT) signaling pathway was especially enriched in epilepsy, which includes signal molecules such as brain-derived neurotrophic factor (BDNF) and neurotrophin-3 (NT3). Previous studies have reported that BDNF is increased in animal models and humans with epilepsy and involved in epileptogenesis [77,78]. Additionally, some pathways were shared in controls and many disorders, such as NGL, CNTN, and CADM, suggesting that these pathways are essential for the maintenance of normal physiological functions and likely do not critically contribute to disease pathogenesis. 

To further investigate the critical disease-related cellular communication in which astrocytes are involved, we compared the significant changed ligand-receptor pairs among different disorders and target cell types (Figure 7D). Specific to NRG and NRXN signaling, we identified two ligand-receptor pairs, NRG3-ERBB4 and NRXN1-NLGN1, as the most significant signaling in epilepsy, contributing to the communication from astrocytes to OPC, Oligo, InN, and ExN. Previous studies reported that NRG3 by non-neuronal cells interacts with ERBB4 in PV interneurons and can promote the formation and maturation of excitatory synapses in the hippocampus [79,80]. We found that NRG3-ERBB4 signaling from astrocytes to OPC, Oligo, and InN widely participated in epilepsy (Figure 7D). In contrast, this pathway had a relatively weak interaction with other diseases, suggesting significant differences in cellular communication among diseases. Together, we found most alterations in cell–cell communications are specific to disorders, identified key signaling changes in epilepsy, and uncovered that astrocytes might actively participate in disease pathogenesis through the perturbation of cellular interactions.

## 4. Discussion

Widespread molecular and functional diversity are recognized to exist in astrocytes across different brain regions and CNS disorders. The development of scRNA-seq enables a high-resolution view of astrocyte heterogeneity, and many studies demonstrated the molecular features of astrocytes in various diseases [1,81,82,83]. For example, Burda et al. and Endo et al. mainly explored astrocyte reactivity in multiple disorders and astrocyte regional diversity based on a large number of mouse models, respectively [82,83]. Especially, Endo et al. identified genes and pathways related to astrocyte morphological complexity that have a significant association with common CNS disorders [83]. Those studies have dramatically changed our previous superficial perception of astrocyte diversity. However, the comprehensive landscape of context-dependent astrocyte reactivity on the human brain is lacking, mainly due to specimen issues, which limit our general understanding of astrocyte transcriptional changes [11]. Here, we integrate the largest human brain scRNA-seq dataset to date, which includes six main neurological diseases. We profiled the astrocytes from three levels, subtype compositions, regulatory modules, and cell–cell communications, which comprehensively reveals the heterogeneity of pathological astrocytes.

We leverage the integrated dataset to better detect novel subpopulations and regulatory modules that could originally not be discovered in small datasets. For example, we identified cluster 3, OLIGO2^+^ astrocyte subpopulations located mainly in white matter. The new subtype highly expressed oligodendrocyte-associated genes. Although the existence of this subtype is controversial in many studies, we have confirmed the possibility of its expression of oligodendrocyte-associated genes from our aspect [5,11]. Through joint analysis with the spatial transcriptomics datasets, we hypothesized that this astrocyte subtype may play an important role in the formation of myelin [47,48]. We also identified an astrocyte subtype, cluster 4, specifically located around the ventricles. This astrocyte subtype may have complex interactions with molecules in the cerebrospinal fluid [10,51]. Single-cell transcriptomics combined with spatial transcriptomics can better complement the spatial information of astrocytes, which can help us further understand the function of astrocytes. We have revealed the spatial specificity of astrocytes, therefore, incorporating the brain region as a key intervention factor of astrocytes is expected to be a promising research direction for treating CNS disorders in the future.

Since we found that the conventional astrocyte composition analysis did not fully explain the occurrence of some diseases, we believe that explaining disease occurrence solely from the perspective of cell subtype abundance or functional modules is insufficient, and a combination of both is necessary. Some diseases may be related to multiple subpopulations, and it is unclear whether these subpopulations collectively contribute to disease occurrence. These factors cannot be explained by a single subpopulation alone, and we must examine them from the perspective of multi-subpopulation functional co-clustering modules. Therefore, we applied NMF analysis to construct seven functional specific co-expression gene modules. The M2 ECM and M4 stress modules showed evident signs of co-regulation, which generated a complex network of interactions. Furthermore, we validated that these two modules were significantly elevated in AD at both the transcriptome and protein levels. Notably, the M2 module was significantly elevated in the early stages of AD onset, which could furnish potential markers for AD early diagnosis. For example, M2 hub protein CD44 has been reported to have the potential as a fluid biomarker for the early diagnosis of AD by a recent proteomic study [84]. 

These astrocyte transcriptomic modules recapitulate the core astrocyte features, which facilitate elucidating diseases mechanisms from a biological perspective. We identified aberration of the M3 synapse and M5 immunity in epilepsy, which are consistent with recent studies [85,86]. The M3 synapse module, which represents the main function of astrocytes, maintains the basic activity of neurons and is also involved in the development of various diseases. A deeper understanding of this pathological process would be very helpful for future research and development of treatments. We also identified the M4 module, which was differentially expressed only in AD. Most importantly, we found, in addition to AD, the M2 ECM module was also elevated in PD. Moreover, our distillation of collectively up- and down-regulated genes among multiple disorders suggests that some critical genes are widely involved in many diseases, such as *MAFG*, *CD44*, and *DCLK1*.

Finally, we performed cellular communication analysis using integrated data and identified disease-specific signaling pathways that could contribute to disease pathogenesis. HD, PD, and Epi disorders correspond to highly disease-specific pathways. Particularly, the NRG3-ERBB4 signaling pathway from astrocytes to InN was significantly elevated in epilepsy. Therefore, we speculate that this signaling pathway of astrocytes may act as a protective function in epileptic pathology and NRG3 may be a new target for anticonvulsant drugs in epilepsy [79,80]. Future studies on the NRG3-ERBB4 pathway in astrocytes and InN could provide richer information on the mechanism of epileptogenesis, which would be an interesting line of research. 

In summary, we integrated published datasets and identified novel and putative functional populations of astrocytes. Future functional studies are needed to evaluate these subpopulations and their potential effectiveness for modulation by therapies. How heterogeneity of astrocytes arises when subjected to different pathogenic stimuli is still an open question. We also hope that continued integration of our current data with future scRNA-seq and spatial datasets will add greater insights into solving these problems and making new discoveries.

## Data Availability

The links and accession ID for public datasets used in this study are listed in Supplementary Table S1.

## Supplementary Materials

The following supporting information can be downloaded at: https://www.mdpi.com/article/10.3390/biom13040692/s1, Supplementary Figure S1: Single-cell RNA-seq dataset quality assessment. (A) Violin plots of gene counts (left panel) and UMI counts (right panel) after quality control filtering. (B) UMAP of integrated data, by dataset of origin. (C) Bar chart summarizing the fraction of different cell types in each dataset source. Supplementary Figure S2: Single-cell RNA-seq dataset quality assessment of astrocytes. (A) Violin plots of gene counts (left panel) and UMI counts (right panel) of astrocyte subtypes. (B) The expression of oligodendrocyte-associated gene (*MBP* and *PLP1*) and tanycyte markers (*COL23A1* and *SLC16A2*) in astrocytes. (C) The correlation analysis between the total MBP expression of each sample’s astrocytes (left panel) and the total *MBP* expression of astrocyte cluster 3 (right panel) and microglia. Each point represents a sample. Correlation coefficients were asserted based on Pearson correlation. Supplementary Figure S3: The unique GO terms of each astrocyte subpopulation. Barplot of the unique GO terms of each astrocyte subtype with the total number of genes involved in each pathway labeled. Supplementary Figure S4: Relative abundance of astrocyte subpopulations across different CNS disorders. Boxplots showing the relative abundance of astrocyte subpopulations in all control and disorder groups as described in (Figure 5A). *p*-values are derived from a two-sided Wilcoxon test. Supplementary Figure S5: Collective and disorder-specific Go terms across different CNS disorders. (A) Boxplots showing the top 10 up-regulated GO terms per disorder. (B) Boxplots showing the top 10 down-regulated GO terms per disorder. (C) GO terms of genes shared by at least three out of six diseases. Red, up-regulated pathways; blue, down-regulated pathways; The dotted line represents *p* < 0.05. Numbers on top of the barplots represent the total number of genes in each pathway. Table S1: Metadata of published datasets used in this study. Table S2: Astrocyte cluster-specific DEGs and GO annotations. Table S3: Gene lists for seven astrocyte expression modules. Table S4: Common and disorder-specific DEGs and Go terms across different CNS disorders.
