# Supplementary figures and images for: Large-Scale Integration of Single-Cell RNA-Seq Data Reveals Astrocyte Diversity and Transcriptomic Modules across Six Central Nervous System Disorders
Source: Biomolecules. 2023 Apr 19;13(4):692. doi: 10.3390/biom13040692 (PMC10135484; doi:10.3390/biom13040692)

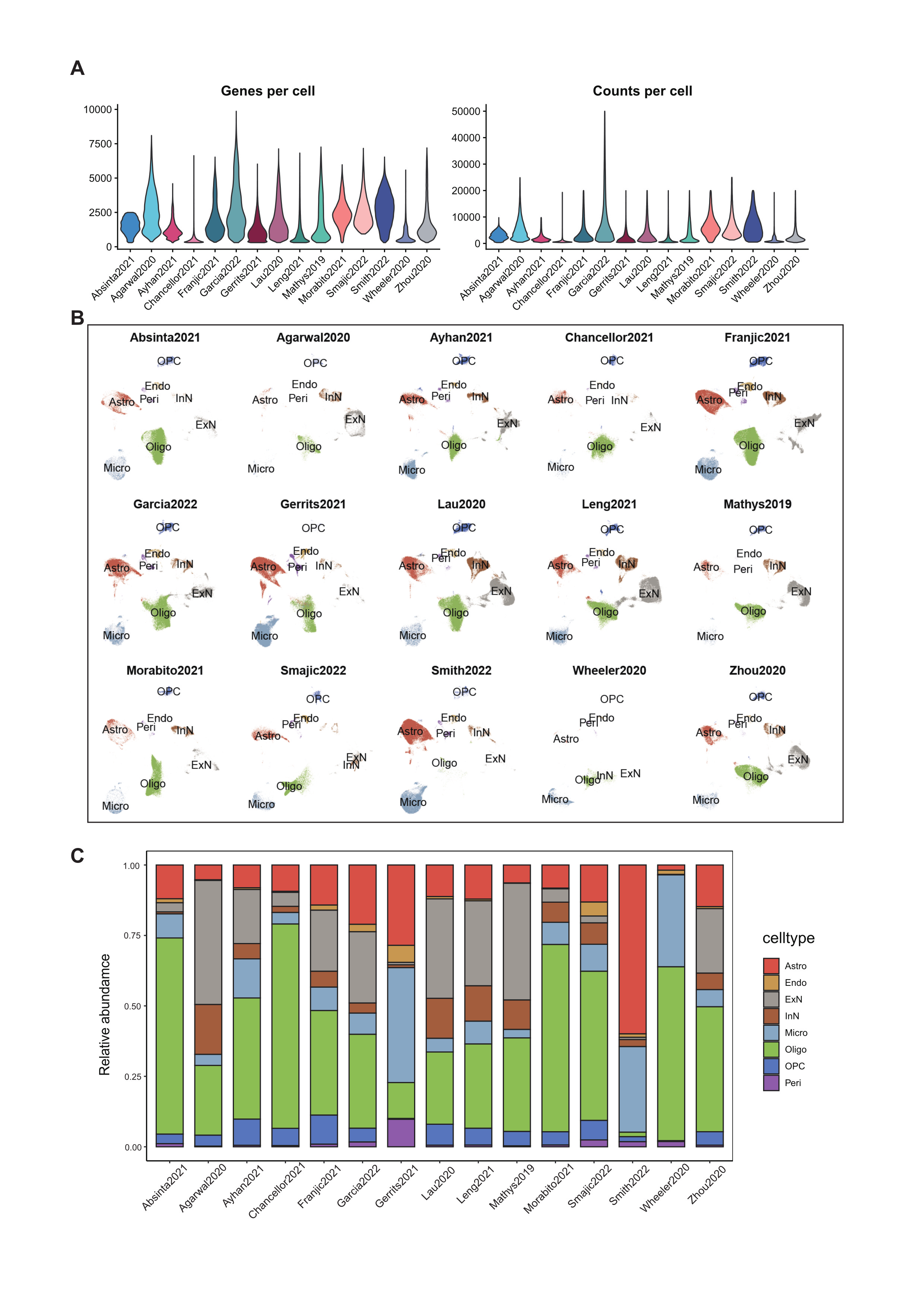

Supplement: Supplementary file 1 [file biomolecules-13-00692-s001.zip › supplementary materials/Figure S1.tif]

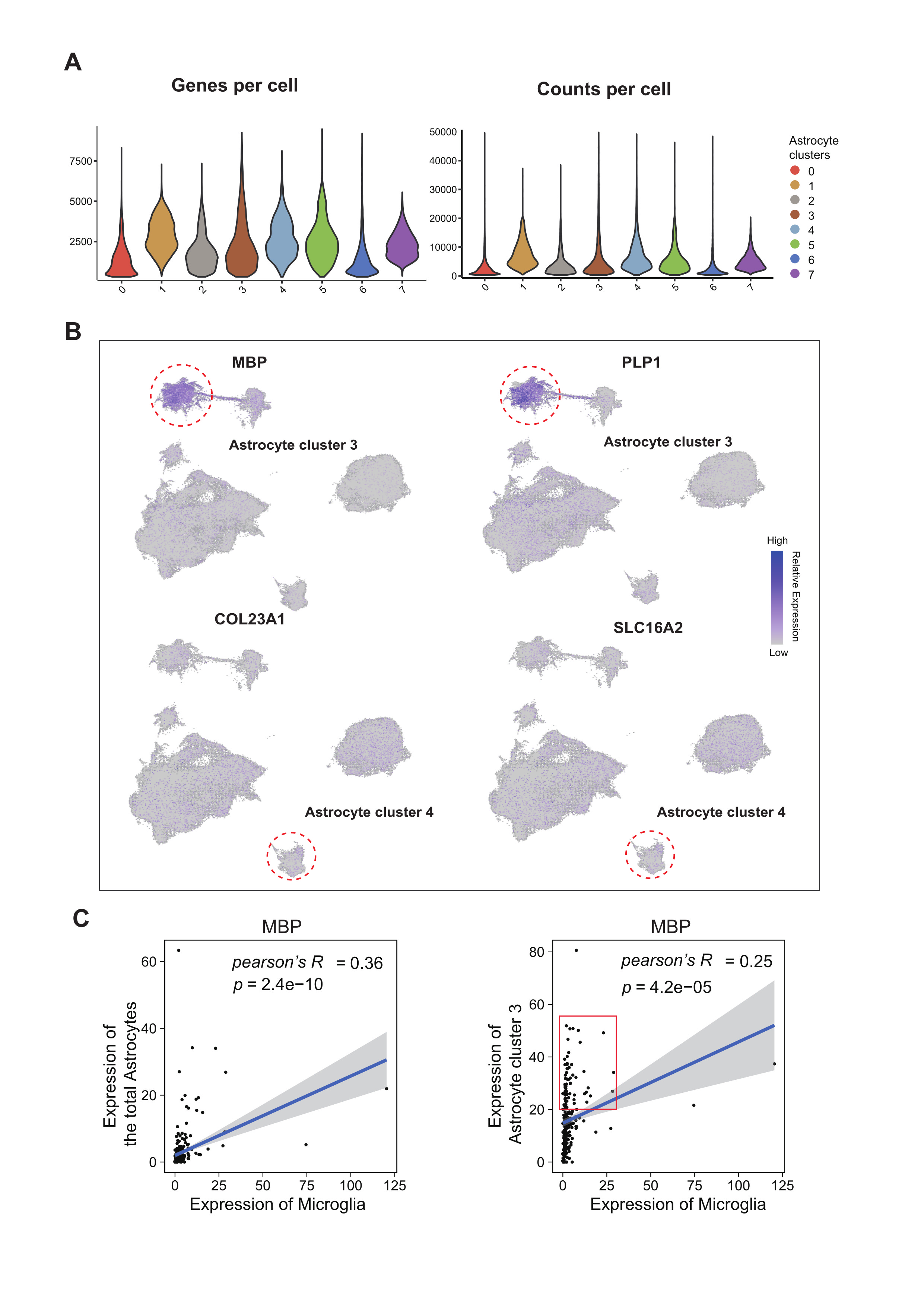

Supplement: Supplementary file 1 [file biomolecules-13-00692-s001.zip › supplementary materials/Figure S2.tif]

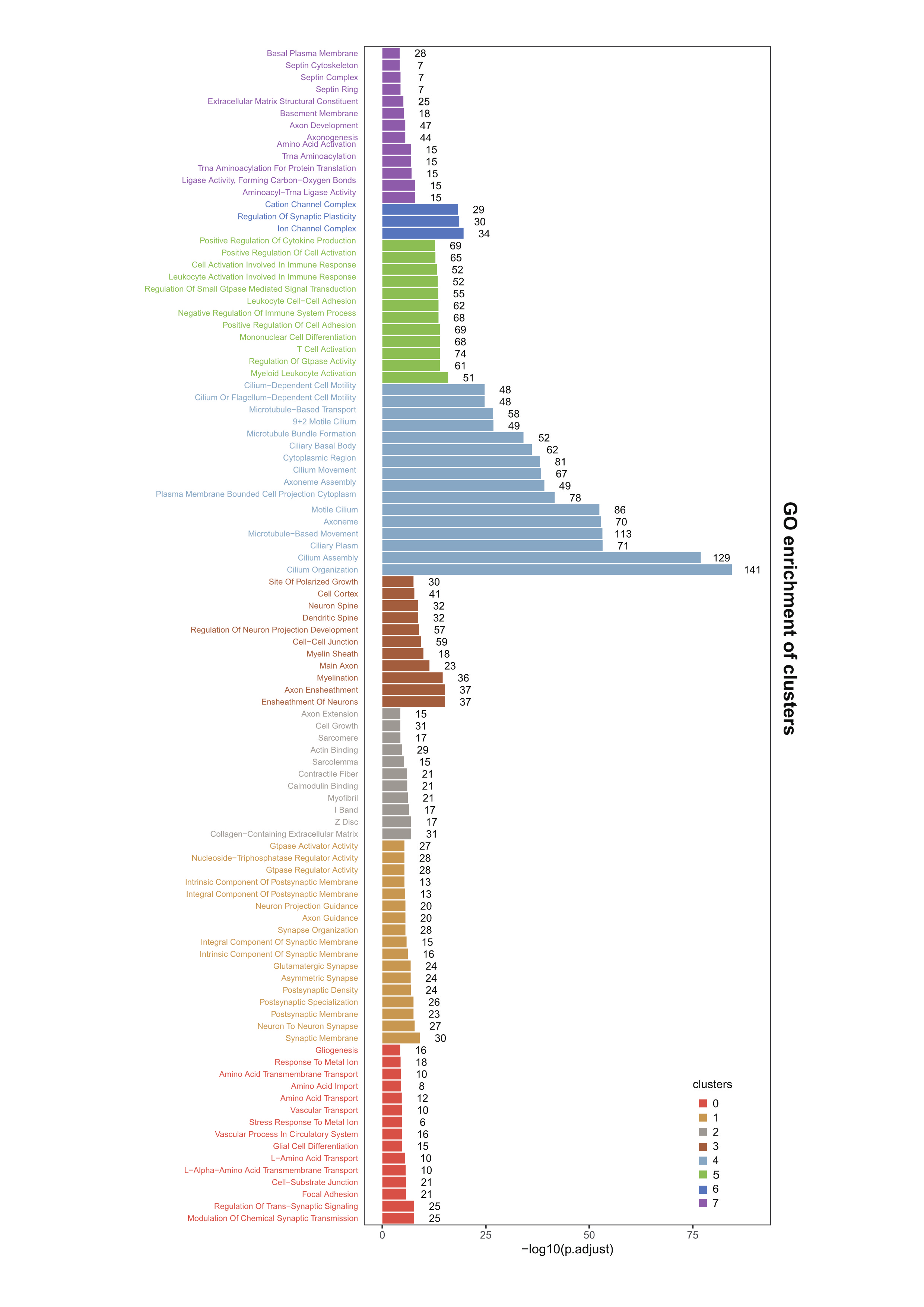

Supplement: Supplementary file 1 [file biomolecules-13-00692-s001.zip › supplementary materials/Figure S3.tif]

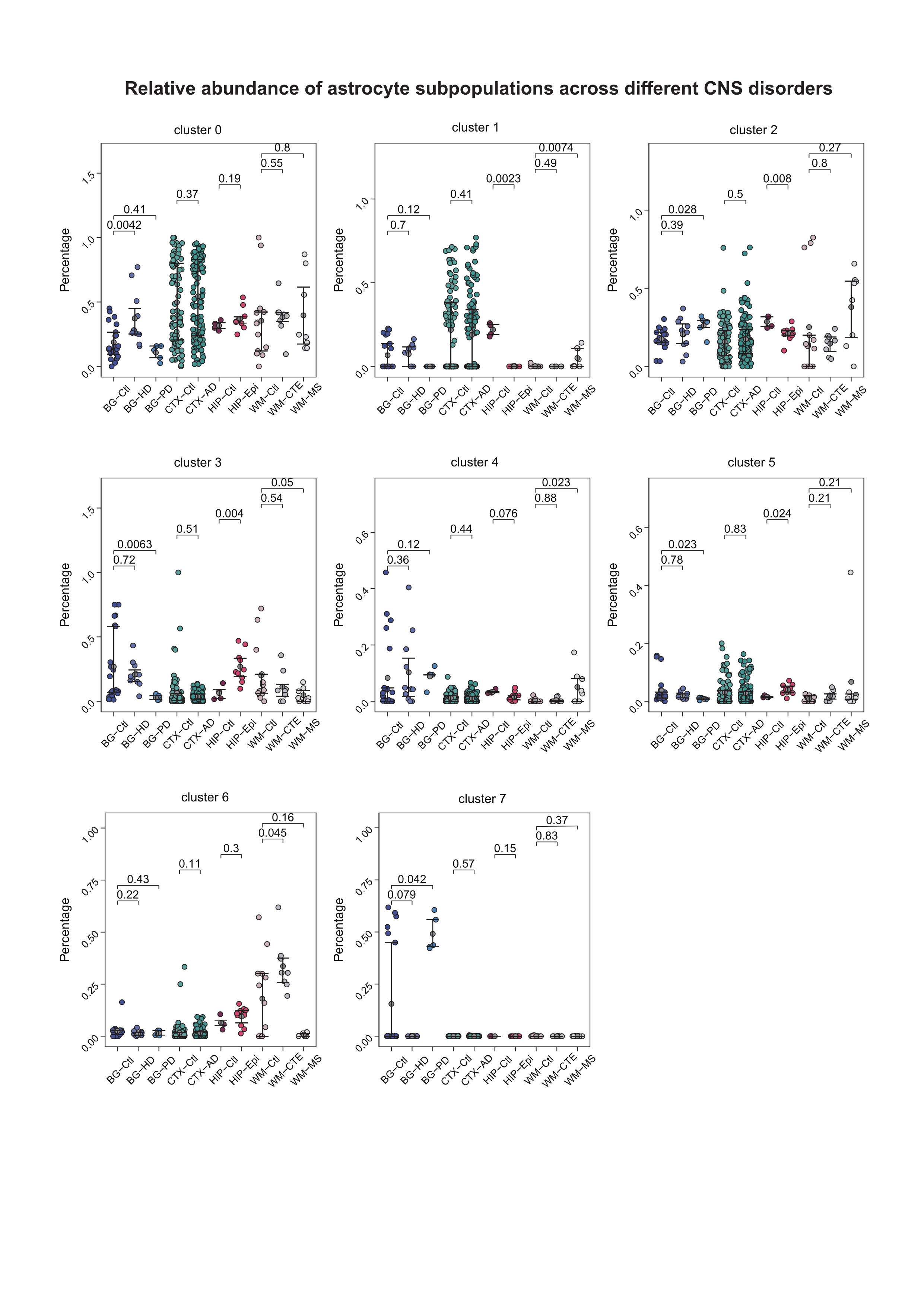

Supplement: Supplementary file 1 [file biomolecules-13-00692-s001.zip › supplementary materials/Figure S4.tif]

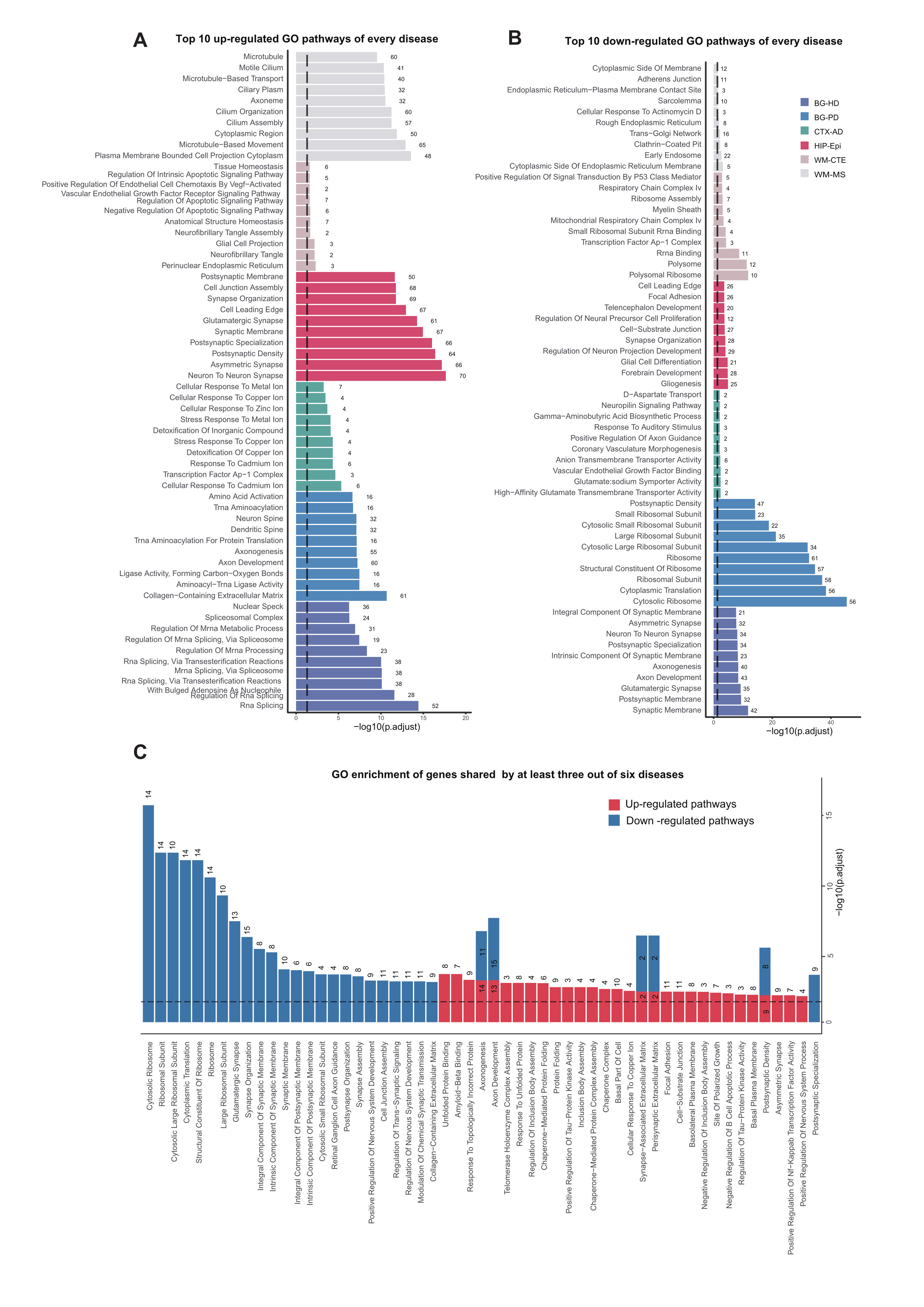

Supplement: Supplementary file 1 [file biomolecules-13-00692-s001.zip › supplementary materials/Figure S5.tif]
